# The Environmental and Social Determinants of Health Matter in a Pandemic: Predictors of COVID-19 Case and Death Rates in New York City

**DOI:** 10.3390/ijerph18168416

**Published:** 2021-08-09

**Authors:** Maria De Jesus, Shalini S. Ramachandra, Zoe Jafflin, Imani Maliti, Aquilah Daughtery, Benjamin Shapiro, William C. Howell, Monica C. Jackson

**Affiliations:** 1 School of International Service and Center on Health, Risk, and Society, American University, Washington, DC 20016, USA; 2 Department of Health Studies, American University, Washington, DC 20016, USA; sr6890a@student.american.edu; 3 Department of Mathematics and Statistics, American University, Washington, DC 20016, USA; whowell@american.edu (W.C.H.); monica@american.edu (M.C.J.); 4 School of International Service, American University, Washington, DC 20016, USA; zj9892a@student.american.edu; 5 Department of Mathematical Sciences, Clark Atlanta University, Atlanta, GA 30314, USA; imani.maliti@gmail.com; 6 Department of Mathematics, Xavier University of Louisiana, New Orleans, LA 70125, USA; adaughte@xula.edu; 7 Department of Computer Science, American University, Washington, DC 20016, USA; bs1572a@american.edu

**Keywords:** COVID-19, coronavirus, death rates, case rates, inequities, New York City, boroughs, social determinants of health, environment, ozone level

## Abstract

Our research objective was to determine which environmental and social factors were predictive of coronavirus disease 2019 (COVID-19) case and death rates in New York City (NYC), the original epicenter of the pandemic in the US, and any differential impacts among the boroughs. Data from various sources on the demographic, health, and environmental characteristics for NYC zip codes, neighborhoods, and boroughs were analyzed along with NYC government’s reported case and death rates by zip code. At the time of analysis, the Bronx had the highest COVID-19 case and death rates, while Manhattan had the lowest rates. Significant predictors of a higher COVID-19 case rate were determined to be proportion of residents aged 65 years plus; proportion of residents under 65 years with a disability; proportion of White residents; proportion of residents without health insurance; number of grocery stores; and a higher ozone level. For COVID-19 death rates, predictors include proportion of residents aged 65 years plus; proportion of residents who are not US citizens; proportion on food stamps; proportion of White residents; proportion of residents under 65 years without health insurance; and a higher level of ozone. Results across boroughs were mixed, which highlights the unique demographic, socioeconomic, and community characteristics of each borough. To reduce COVID-19 inequities, it is vital that the NYC government center the environmental and social determinants of health in policies and community-engaged interventions adapted to each borough.

## 1. Introduction

**Figure 1:**
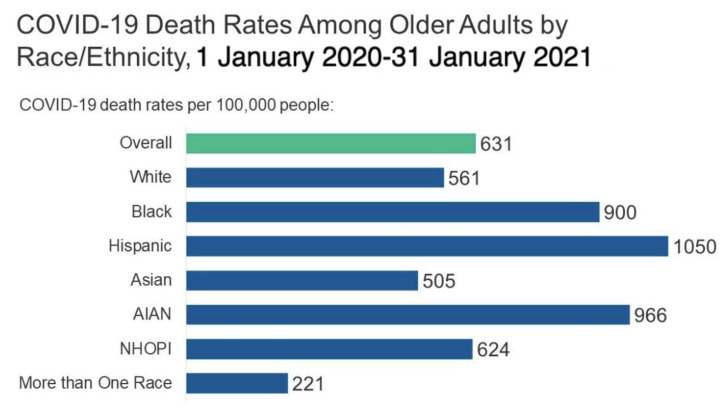
Disproportionate coronavirus disease 2019 (COVID-19) mortality rates. AIAN: American Indian and Alaska Native; NHOPI: Native Hawaiian and Other Pacific Islanders.

Approximately 19 months into the coronavirus disease 2019 (COVID-19) ongoing pandemic, the death toll amounts to approximately 607,000 individuals in the United States (US) alone and 4.26 million individuals globally [1,2]. Since the beginning of the pandemic in January 2020, the cumulative number of confirmed cases in the US at the end of July 2021 is 34.53 million and 194.72 million worldwide [3]. In addition, it is increasingly clear that social and health inequities are profoundly, and unevenly, impacting COVID-19 morbidity and mortality. The most pervasive inequities are observed among Black, Latinx, and American Indian and Alaska Native (AIAN) populations, with COVID-19 death rates that are more than 1.5 times the rate among older White adults (Figure 1) [4].

Similarly, recent data from the Centers for Disease Control and Prevention (CDC) demonstrate a consistent pattern of racial/ethnic differences in the risk for COVID-19 infection, hospitalization, and death (Table 1) [5,6,7].

### 1.1. Social Determinants of Health and COVID-19 Exposure

**Figure 2:**
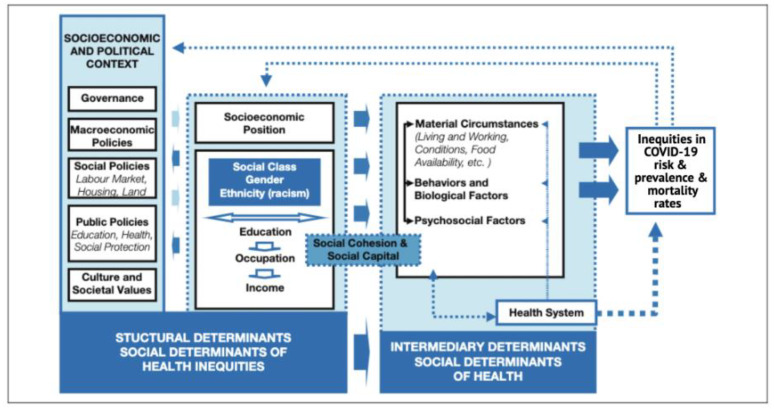
Adapted conceptual framework for action on the social determinants of health, 2010. Data source: World Health Organization [8].

Social determinants of health (SDH) have been conceptualized as the drivers of health for decades by many public health experts and are often at the root of health inequities. COVID-19 has brought to light many of these long-existing inequities [8]. Race and ethnicity are risk markers for the SDH, including socioeconomic status, access to health care, healthy living and working conditions, and exposure to the virus related to occupation (e.g., frontline, essential, and critical infrastructure workers). These upstream determinants help explain why race/ethnicity is associated with differential risks for COVID-19 infection and mortality. They also create social stratification by income, education, class, gender, and race/ethnicity, including those associated with COVID-19. The SDH framework encompasses social, economic, and political systems and structural mechanisms that drive inequities in COVID-19 risk, and prevalence and mortality rates (Figure 2).

Adopting the SDH framework, the study seeks to examine which environmental and social factors were most predictive of COVID-19 case and death rates in New York City (NYC), the original epicenter of the pandemic in the US, and any differential impacts among the boroughs. The first case relating to the COVID-19 pandemic in the United States was confirmed in NYC on 29 February 2020 [9]. Approximately one month later, the metropolitan area was the worst-affected area in the country, with its medical infrastructure overburdened [10,11]). Since then, COVID-19 continued to spread across the city; by 5 August 2021, the cumulative number of cases was 982,642 and cumulative deaths was 33,570 [12]. New York City is composed of five boroughs (the Bronx, Brooklyn, Manhattan, Queens, and Staten Island), each with unique demographic, socioeconomic, and community characteristics. Understanding the factors that contribute to the COVID-19 local case and death rates in NYC across the different boroughs has implications for the development of evidence-based pandemic public health planning and policy and interventions that are adapted for local contexts and community characteristics. These results are needed to inform future approaches to mitigate a possible resurgence of the disease, in particular as new variants of COVID-19 continue to emerge. Numerous studies have demonstrated that certain social determinants of health, such as poverty, lack of healthcare access, overcrowded housing, place of residence, and toxic physical exposures (e.g., smoke inhalation, air pollution), are key determinants in the transmission of COVID-19 [13,14,15,16]. Having a low-income, work-related job, such as in the service sector, also increases the likelihood of contracting COVID-19, as these jobs may require the use of public transportation and high physical proximity to others [13,17]. Service and industrial sector jobs also do not typically accommodate shelter-in-place or “stay at home” alternatives, opening the risk of unemployment or reduced income. These types of jobs are most often occupied by low-income ethnic-racial minority populations, which automatically puts them at a disproportionate risk to exposure [18].

### 1.2. The Role of the Living and Working Conditions in COVID-19 Cases and Death Rates

Physical distancing measures, which are necessary to prevent the spread of COVID-19, are substantially more difficult for those with adverse social determinants, contributing to higher rates of COVID-19 morbidity and mortality. As Yancy CW states, “social distancing is a privilege” that is simply not accessible in some communities, and the ability to isolate in one’s home and have full digital access to technology and the Internet are components of that privilege [6]. People in low-income jobs may also delay seeking care for COVID-19, fearing high hospital fees or full exposure from a high-contact hospital, which could potentially result in loss of income/unemployment, more severe illness, and poorer mental health outcomes [13]. Living in crowded conditions and having limited material circumstances (e.g., overcrowded households, homelessness, etc.) can also increase the likelihood of airway infections according to 2018 World Health Organization (WHO) reports, which, due to the pandemic’s air-borne nature, is applicable to COVID-19 [13].

Individuals living in these adverse living and working conditions are more likely to experience underlying medical conditions, such as asthma and obesity, which in turn, places them at higher risk for severe cases of COVID-19 [19,20]. For example, national current asthma prevalence data reveal that Puerto Rican (14%), American Indian/Native American (12%), and Black (11%) populations have the highest current rates compared to other racial/ethnic groups (8% among White, 6% among Latinx overall, and 4% among Asian populations) [21]. Low-income households (i.e., those below 100% of the poverty threshold) also had the highest rate (12%) of asthma compared to other socioeconomic groups [21].

### 1.3. A Crisis within a Crisis: Environmental Injustice and the COVID-19 Pandemic

Environmental injustice is experienced through heightened exposure to air pollution and associated health risks, limited access to adequate environmental services (e.g., waste removal services, landscape maintenance, and sanitation assistance), and loss of land or resource rights [22]. A key determinant of health, as mentioned above, is the physical environment [23,24]. The changing demographics of urban areas due to urbanization/de-urbanization, rise of megacities, and other changes to population characteristics have contributed to a disproportionate number of members of ethnically/racially diverse populations residing in concentrated areas [22]. Poor and loose permitting requirements (especially those for land use and manufacturing) along with exclusionary zoning laws add to the already high degree of environmental degradation and reduced social support in large cities, increasing the burden communities of low socioeconomic background face every day.

On average, members of ethnically/racially diverse populations comprise 56% of the population living in neighborhoods with Toxic Release Inventory (TRI) facilities in place (i.e., programs providing information regarding toxic chemical releases and waste management activities in their communities) compared to 30% elsewhere [25]. Such facilities emphasize how negative environmental factors, such as living next to a waste disposal site, can compound social and economic conditions and lead to higher levels of chronic health problems, such as asthma, diabetes, and hypertension, for ethnically/racially diverse and low-income populations [26]. Astell-Burt et al. (2013) examined the effects of air pollution on racism and ethnicity in adolescents living in an urban environment and found no genetic predispositions to chronic respiratory illnesses such as asthma based on ethnicity, only environmental racism [27].

Specifically, for asthma, a link between air pollutants and SARS-CoV-2 is considered to create a “double-hit” to the lungs leading to acute lung injury by attenuating tissue remodeling and influencing local inflammatory response [28]. In a recent Italian study, chronic exposure to air pollution was found to be an accelerator of COVID-19, and the highest number of COVID-19 cases were recorded in the most polluted regions in Italy, with patients presenting with more severe forms of the disease requiring intensive care unit (ICU) admission [28]. In these regions, mortality was also two-fold higher than in the other regions [28]. With an already susceptible respiratory system, individuals who suffer from asthma and who are chronically exposed to air pollutants are more likely to experience a severe form of SARS-CoV-2, short-term and long-term respiratory complications, and/or hospitalization from COVID-19 [28]. Other environmental studies also found a positive relationship between air pollutants and COVID-19 outcomes in several US counties, England, and China [29,30,31].

In another study, based on spatial data from the Bronx in NYC, a concentrated urban environment, Maantay (2007) found that air pollutants led to high asthma hospitalization rates, which particularly affected members of ethnic/racial minority groups and those living in poverty [32]. Individuals living near noxious land uses were up to 66% more likely to be hospitalized for asthma and were 30% more likely to be poor and 13% more likely to be a member of an ethnic/racial group [32]. This NYC-based study was conducted prior to the COVID-19 pandemic. Thus, the links between air pollutants and other key determinants of health, race/ethnicity, poverty, and COVID-19 warrant further analysis. Our study will, therefore, fill in this gap by examining the relationship between the physical environment, namely the level of ozone and fine particulate matter in the air, and other key social determinants of health (such as household income, poverty, ethnicity/race, age, education level, health insurance status, obesity, number of asthma-related emergency room visits, overcrowding, citizenship status, receipt of food stamps, a US federal program that provides food-purchasing assistance for low- and no-income people, etc.) and COVID-19 case and death rates in NYC. Guided by the social determinants of health framework, we selected potential variables captured in reliable data sources that may have influenced the COVID-19 case and death rate in NYC, as described in detail below.

## 2. Materials and Methods

### 2.1. Data

We analyzed secondary data on various demographic, health, and environmental characteristics for NYC zip codes, neighborhoods, and boroughs along with the NYC government’s reported case and death rates by zip code (see details below). Table 2 lists all the variables used in this study.

The New York City Health Department provided data on the COVID-19 case and death rates by zip code, each of which belongs to one of five boroughs: the Bronx, Brooklyn, Manhattan, Queens, and Staten Island (City of New York 2020a) [33]. The Statistical Atlas (2018), extracting data from the 2010 Census Bureau and the 2012–2016 American Community Survey, provided demographic information of the residents per zip code (i.e., percent of 65 years and older; self-identify as White; are non-US citizens; receive food stamps; without a high school diploma; and median household income) [34]. The NYC Government Environment and Health Data Portal provided data on six variables: number of adult asthma emergency room (ER) visits; level of fine particulate matter in the air; ozone level; percent who are obese; percent of homes that are overcrowded; and percent of poverty (City of New York 2020b) [35]. 

Values were listed per neighborhood, which encompassed certain zip codes. Five zip codes used in NYC’s COVID-19 data were not listed in this portal (10065, 10075, 10069, 10282, and 11109), therefore, we assigned them to appropriate neighborhoods. The United States Census Bureau (2019) supplied data for percent of residents under 65 years old who have a disability [36]. The United States Department of Agriculture’s Food Environment Atlas (2019) supplied county level data for the variables: number of grocery stores per 1000 residents and percent of residents under 65 years old without health insurance [37]. The Centers for Disease Control and Prevention COVID Data Tracker (2020) provided the transportation variables of mobility index or how far the median user in each county moves per day and percent of reduction in travel to transit stations [38]. The variables were collected from five counties of New York City (i.e., Bronx County, New York County, Kings County, Queens County, and Richmond County), each of which encompasses one of the five boroughs. Table 3 provides the numerical characteristics of New York City for all quantitative variables.

### 2.2. Statistical and Graphical Analysis

Pearson correlation was used to measure the correlation between all variables (Table 4). Explanatory variables were removed from later regression models if their correlation with another explanatory variable was at *r* = ±0.8 or more extreme or slightly less if an exception to reduce confounding. For all correlation tests with either response variable COVID-19 case rate or COVID-19 death rate, strong correlations were considered to have an *r* value of ±0.75 or more extreme. Multiple regressions analyses were used to predict NYC COVID-19 case rate and COVID-19 death rate, non-stratified and stratified by borough. 

The significance levels α = 0.05 and α = 0.10 were noted for correlation tests, while the significance level α = 0.10 was used for the regression analysis. Given that this was an exploratory study guided by the social determinants of health framework, we wanted to examine the potential variables that may have influenced the case and death rate in a comprehensive manner. All assumptions were reasonably met for every test and all analyses were completed with the use of RStudio [39]. Maps were created with the use of the ArcGIS online software and a borough boundaries layer provided by the NYC government (ESRI 2011, West Redlands, CA, USA; NYC Open Data 2020). 

## 3. Results

### 3.1. Maps of COVID-19 Case and Death Rates

**Figure 3:**
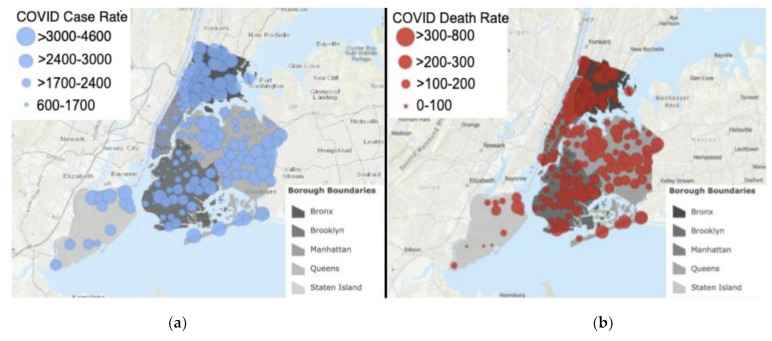
(**a**) Map of COVID-19 case rate (per 100,000 residents) in New York City per zip code with shaded borough boundaries; (**b**) Map of COVID-19 death rate (per 100,000 residents) in New York City per zip code with shaded borough boundaries.

Figure 3 depicts the COVID-19 case rates and death rates. The Bronx has the highest COVID-19 case and death rates, while Manhattan has the lowest rates.

### 3.2. Correlation Matrix of All Explanatory Variables and Response Variables

Based on Table 4, neither COVID-19 case rate nor COVID-19 death rate is strongly correlated with any explanatory variables at *r* = ±0.75 or more extreme.

The variable percent in poverty was highly correlated with a few explanatory variables, such as percent on food stamps (*r* = 0.817), percent without a high school diploma (*r* = 0.728), and adult asthma ER visits (*r* = 0.826), leading us to exclude percent in poverty from the regression analysis due to confounding. Because fine particulate matter level and ozone level were highly correlated (*r* = −0.881), we chose to remove fine particulate matter level for the regression analysis. Because ozone level and mobility index were highly correlated (*r* = 0.747), and mobility index was also a similar measurement to percent decrease transit that would cause singularity issues in later regression models, we also chose to remove mobility index from subsequent regression models, even though the correlation coefficient did not reach our threshold of 0.8. Median household income, percent on food stamps, and percent without a high school diploma were also highly correlated with one another, so we chose to run separate regressions, one with each of the three variables as well as the rest of the explanatory variables to select the best fit model.

### 3.3. Multiple Regression, Non-Stratified and Stratified by NYC Borough with COVID-19 Case Rate

**Figure 4:**
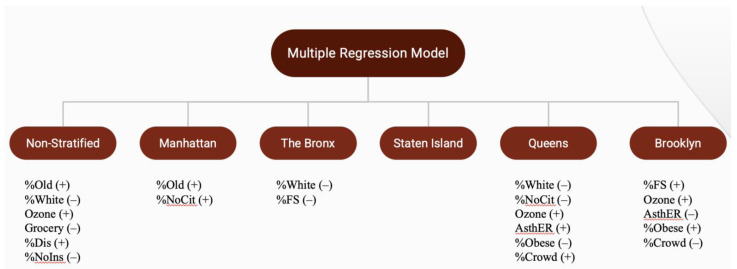
Summary of multiple regression of New York City COVID-19 case rate.

Table 5 and Figure 4 summarize the results of a multiple regression, non-stratified and stratified by NYC borough with COVID-19 case rate as the response variable. Based on the correlation results, certain variables were excluded to reduce confounding effects and multicollinearity. Out of the three models containing either median household income, percent on food stamps, or percent without high school diploma, the model with percent on food stamps had the highest adjusted *R*-squared and lowest Akaike's Information Criteria(AIC) and Bayesian Information Criteria (BIC), leading us to choose the model with percent on food stamps as the best fit to predict COVID-19 case rate.

The overall model was significant, with a *p*-value less than 0.001. Approximately 65.65% of the variability in the model is explained by the factors included. Significant predictors of NYC COVID-19 case rate include percent 65+, percent White, ozone level, number of grocery stores, percent Disability, and percent without health insurance.

For Manhattan, approximately 67.84% of the variability in the model is explained by the factors included, and the model was significant (*p* < 0.001). Percent 65+ and percent non-US citizen were statistically significant variables in predicting the COVID-19 case rate of this borough. 

In the Staten Island model, none of the individual explanatory variables were significant. The model overall was significant (*p* = 0.0682), and approximately 72.09% of the variability in the model is explained by the factors included.

The Bronx model was significant (*p* = 0.0764), but only approximately 29.91% of the variability in the model is explained by the factors included. Significant predictors for the Bronx model include percent White and percent on food stamps.

In the Queens model, approximately 58.8% of the variability in the model is explained by the factors included, and the model was significant (*p* < 0.001). The significant variables are percent White, percent non-US citizen, ozone level, adult asthma ER visits, percent obese, and percent overcrowding.

For Brooklyn, percent on food stamps, ozone level, adult asthma ER visits, percent obese, and percent overcrowding significantly predicted the borough’s COVID-19 case rate. The model was significant (*p* < 0.001), and approximately 71.77% of the variability in the model was explained by the factors included.

### 3.4. Multiple Regression, Non-Stratified and Stratified by NYC Borough with COVID-19 Death Rate

**Figure 5:**
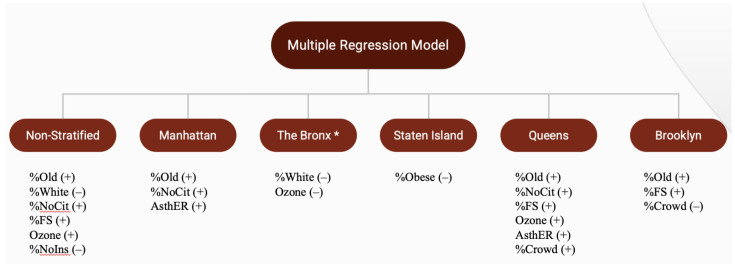
Summary of multiple regression of New York City COVID-19 death rate.

Table 6 and Figure 5 summarize the results of a multiple regression, non-stratified and stratified by NYC borough with COVID-19 death rate as the response variable. Based on the correlation results, certain variables were excluded to reduce confounding effects and multicollinearity. Out of the three models containing either median household income, percent on food stamps, or percent without high school diploma, the model with percent on food stamps had the highest adjusted *R*-squared and lowest AIC and BIC, leading us to choose the model with percent on food stamps as the best fit to predict COVID-19 death rate. 

The overall model was significant, with a *p*-value less than 0.001. Approximately 54.84% of the variability in the model is explained by the factors included. Significant predictors of COVID-19 death rate included percent 65+, percent White, percent non-US citizen, percent on food stamps, ozone level, and percent without health insurance.

For Manhattan, percent 65+, percent non-US citizen, and adult asthma ER visits were significant predictors of the COVID-19 death rate in that borough. The model was significant (*p* < 0.001), and approximately 64.33% of the variability in the model is explained by the factors included.

The only significant predictor for Staten Island was percent obese. For this borough, the model was also significant (*p* = 0.045), and approximately 77.79% of the variability in the model is explained by the included factors.

The Bronx model was not significant (*p* = 0.1038). Approximately 26.14% of the variability in this model is explained by the included factors. The variables percent White and ozone level were significant predictors for the Bronx’s COVID-19 death rate. 

The model for Queens was significant (*p* < 0.001), and approximately 46.63% of the variability in the model is explained by the factors included. Percent 65+, percent non-US citizen, percent on food stamps, ozone level, and adult asthma ER visits were all significant predictors of the borough’s COVID-19 death rate. 

For the Brooklyn borough, the model was also significant overall (*p* < 0.001), and approximately 83.15% of the variability in the model is explained by the included factors. The significant predictors of Brooklyn’s COVID-19 death rate were percent 65+, percent on food stamps, and percent overcrowding.

## 4. Discussion

Consistent with the national pattern of racial/ethnic differences in the risk for COVID-19 infection and death, our maps (Figure 3) [5,6,7] also indicate a similar pattern of inequities among the boroughs. It is important to interpret these findings in light of the social determinants of health [8] in order to understand the factors that are contributing to these inequitable rates across the boroughs. The Bronx, which has the highest proportion of members of racial/ethnic groups, the highest number of people living in poverty, and the lowest levels of educational attainment, had higher rates of COVID-19 case and death rates than the other four boroughs. In contrast, the COVID-19 case and death rates were lowest among residents of the most affluent borough, Manhattan, which is composed of a predominately White population. It is important to note that Manhattan and the Bronx have the highest number of per capita hospital beds, and Manhattan has the highest population density, which suggests that other factors, such as underlying comorbid illnesses, occupational exposures, and socioeconomic and racial/ethnic-based structural inequities explain the differential outcomes among the boroughs. 

Our results of the overall non-stratified multiple regression of predicting NYC COVID-19 case and death rates were in line with previous studies [5,6,13,14,16] related to the environmental and social determinants of health driving a disproportionate burden of COVID-19 morbidity and mortality. Results of the significant predictors of the COVID-19 case and death rates for each borough were mixed, which is likely due to the unique demographic, socioeconomic, and community characteristics of each borough. The significant predictors of COVID-19 case rate in the Bronx were percent of White residents and percent of residents on food stamps (proxy for poverty), while the percent of White residents and ozone level predicted COVID-19 death rates. These results are meaningful when one considers the characteristics of this particular borough. The Bronx has a disproportionately higher rate of people living in poverty (26.2%), higher than the overall rate of NYC (17.9%) and every other borough (Brooklyn: 17.7%; Manhattan: 14.1%; Queens: 11.0%; and Staten Island: 9.1%) [36]. In addition, the Bronx is comprised of the highest percent of non-White populations, with a majority Black (43.6%) and Latinx (56.4%) population compared to other boroughs [36]. It also has the highest percent of individuals who speak a language other than English at home (59.3%) compared to the other boroughs (Brooklyn: 45.0%; Manhattan: 39.4%; Queens: 55.7%; and Staten Island: 32.6%) and the lowest rate of individuals with a university degree (20.1%) compared to Brooklyn (37.5%), Manhattan (61.3%), Queens (32.2%), and Staten Island (33.9%) [36].

Within the case rate models, specific patterns emerged among which explanatory variables were significant. Consistent with the literature [13,14], ethnicity/race and age were important predictors. Other determinants such as the number of grocery stores and percent with a disability were also significant, which is justifiable because both factors lead to situations where individuals need to come in contact with people due to reliance on public transit or dependence on daily assistance. More interestingly, a higher ozone level in the atmosphere predicted a higher case rate in multiple models. Poorer neighborhoods in NYC are more likely to also have poorer air quality, as evidenced by higher levels of mean ppb in ozone, a common air pollutant that can harm breathing and worsen asthma and other respiratory conditions [40]. These findings are supported by previous studies [29,30,31] of a positive relationship between the concentration of air pollutants and COVID-19 cases. Tailoring the COVID-19 response in a way that mitigates poor air quality and provides additional resources to boroughs with worse air quality may successfully lessen morbidity and mortality rates. Additionally, further examination of the relationship between air pollutants and COVID- 19 and possibly other respiratory diseases across different regions would be a fruitful potential direction for this research.

Other results in relation to the COVID-19 case and death rate models are counter-intuitive and warrant further research. For example, the proportion of crowded households had a positive standardized estimate for Queens and a negative standardized estimate for Brooklyn for both case and death rates. This latter finding suggests that the higher the proportion of crowded households in Brooklyn was, the lower the COVID-19 case and death rates would be. These results are unexpected given that close contact is a major mechanism for spreading the virus. However, it is important to note that housing safety protocols in NYC during the pandemic were not studied; thus, crowded housing warrants further investigation. Another possible explanation for these counter-intuitive results is the definition of “overcrowding” used by the New York City Department of Housing Preservation and Development (HPD), which considers “dwelling units occupied by more than one person per room as crowded” [35].

There were no significant variables for the Staten Island case rate model, and only one variable (proportion of obese residents) was significant in predicting COVID-19 death rates, which suggests that other factors not included in our study and unique to Staten Island should be examined. Surprisingly, based on the non-stratified COVID-19 case and death rate models, a higher proportion of residents without health insurance predicted lower case and death rates. One explanation may be the accessibility of COVID-19 testing. It is worth noting that according to the NYC COVID-19 Citywide Information Portal [35], one is not required to have insurance to receive a diagnostic test. Another explanation may be that the data for percent without insurance were collected per borough and not more specifically, such as by zip code or neighborhood, which may be a study limitation that influenced the results

Collectively, our results can be interpreted in light of the environmental and social determinants that drive observed COVID-19 outcomes in NYC and in specific boroughs. Higher COVID-19 case and death rates in the Bronx points to inequities in terms of a disproportionate number of people who are living and working in contexts of higher risk (e.g., poorer neighborhoods with poorer air quality, overcrowded living arrangements, and frontline workers like taxi drivers and service and industrial sector workers, who are more likely to get infected with the coronavirus and die from COVID-19). It is, therefore, important to understand the patterns of poverty level, racial/ethnic residential composition, education, air quality, and other relevant environmental and social determinants of health in each borough for the local and federal governments to figure out how and where to allocate more resources and craft policies to mitigate the worse COVID-19 case and death outcomes.

### 4.1. Limitations

When studying COVID-19, especially in the US, where information is continuously being updated, there are a few limitations. It is difficult to measure human behaviors such as individuals not following safety guidelines—whether due to the lack of knowledge at the beginning of the outbreak, the assortment of conspiracy theories, or other reasons—that may be influencing the case and death rates. Other limitations include not having a measurement for the reliability of test results, taking into consideration false positives and negatives, as well as not being able to factor in the general accessibility of getting tested. Despite these limitations, the study contributes to the literature on the importance of implementing an approach that takes into account and addresses the social and physical determinants of health to mitigate COVID-19 outcomes. 

### 4.2. Future Research Directions and Policy and Intervention Implications

Future research studies should analyze which of NYC actions were actually successful or harmful in addressing the COVID-19 pandemic. Studies are also needed to investigate the short-term and long-term effects of COVID-19 on health and how differential outcomes can be reduced in anticipation of subsequent waves of cases, as new variants of the virus emerge. Study results demonstrate that addressing the environmental and social determinants of health must be an essential part of a comprehensive response to the COVID-19 pandemic. The higher rates of morbidity and mortality for COVID-19 observed among racial/ethnic minority populations are, in large part, a product of the physical conditions in which individuals live and work.

## 5. Conclusions

Public policies have the power to enhance health and also exacerbate COVID-19 inequities. With these analyses, we aimed to shed light on the related underlying environmental and social determinants of health related to COVID-19 case and death rates as well as how the local government can focus its COVID-19 response efforts towards the most impactful factors in order to reduce the case and death rates in the city and its boroughs. Going forward, the New York City government should focus on reducing health inequities, including COVID-19 inequities, by putting efforts towards centering the environmental and social determinants of health in policies [8] and community-engaged interventions. In addition, it is vital to properly allocate the necessary resources adapted for local contexts and boroughs in order to mitigate the COVID-19 case and death rates. Such approaches require a deep understanding of community, consideration of local data-driven approaches, diverse and equitable partnerships across sectors, and policies that reduce COVID-19 inequities.

## Figures and Tables

**Table 1:** Risk for COVID-19 infection, hospitalization, and death by race/ethnicity.

**Rate Ratios Compared to White, Non-Hispanic Persons**	**American Indian or Alaska Native, Non-Hispanic Persons**	**Asian, Non-Hispanic Persons**	**Black or African American, Non-Hispanic Persons**	**Hispanic or Latino Persons**
**Cases ^1^**	1.6x	0.7x	1.1x	2.0x
**Hospitalization ^2^**	3.3x	1.0x	2.9x	2.8x
**Death ^3^**	2.4x	1.0x	2.0x	2.3x

^1^ Data source: Data reported by state and territorial jurisdictions (accessed on 9 June 2021). Numbers are ratios of age-adjusted rates standardized to the 2019 US intercensal population estimate. Calculations use only the 61% of case reports that have race and ethnicity; this can result in inaccurate estimates of the relative risk among groups; ^2^ Data source: COVID-NET (https://www.cdc.gov/coronavirus/2019-ncov/covid-data/covid-net/purpose-methods.html, accessed on 1 March 2020 and 1 June 2021). Numbers are ratios of age-adjusted rates standardized to the 2019 US standard COVID-NET catchment population; ^3^ Data source: National Center for Health Statistics (NCHS) provisional death counts (https://data.cdc.gov/NCHS/Provisional-Death-Counts-for-Coronavirus-Disease-C/pj7m-y5uh, accessed on 5 June 2021). Numbers are ratios of age-adjusted rates standardized to the 2019 US intercensal population estimate.

**Table 2:** Variable names and descriptions.

Variable Name	Variable Label	Description
CaseR	COVID-19 case rate	Number of New York City COVID-19 cases per 100,000 residents, downloaded on 2 July 2020
DeathR	COVID-19 death rate	Number of New York City COVID-19 deaths per 100,000 residents, downloaded on 2 July 2020
%Old	Percent 65+	Percent of residents aged 65 and older, measured in the 2010 Census Data and the 2012–2016 American Community Survey
%White	Percent White	Percent of residents who are White, measured in the 2010 Census Data and the 2012–2016 American Community Survey
%NoCit	Percent non-US citizen	Percent of residents who are not US citizens, measured in the 2010 Census Data and the 2012–2016 American Community Survey
Income	Median household income	Median household income of residents, measured in the 2010 Census Data and the 2012–2016 American Community Survey
%FS	Percent on food stamps	Percent of residents on food stamps, measured in the 2010 Census Data and the 2012–2016 American Community Survey
%NoHS	Percent without high school diploma	Percent of residents with no high school diploma, measured in the 2010 Census Data and the 2012–2016 American Community Survey
AsthER	Adult asthma ER visits	Number of adult asthma emergency room (ER) visits (age-adjusted per 10,000 residents), measured in 2016
FPM	Fine particulate matter level	Fine particulate matter level (mcg/m^3^), measured in 2018
Ozone	Ozone level	Level of ozone (ppb), measured in 2018
%Obese	Percent obese	Proportion of residents who are obese, measured in 2017
%Crowd	Percent overcrowding	Proportion of homes with >1 person per room, measured from 2013 to 2017
%Pov	Percent in poverty	Proportion of residents in poverty, measured from 2013 to 2017
Grocery	Number of grocery stores	Number of grocery stores (per 1000 residents) measured in 2014
%Dis	Percent disability	Proportion of residents under 65 years old who have a disability, measured from 2014–2018
%NoIns	Percent without insurance	Proportion of residents under 65 years old with no health insurance, measured from 2014–2018
Mobility	Mobility index	Mobility index of how far the median user in each county moves per day, measured on 30 June 2020
%DecT	Percent decrease transit	Proportion reduction in travel to transit stations, measured on 30 June 2020

**Table 3:** Numerical characteristics of New York City for all quantitative variables.

	Minimum	Maximum	Median	Mean	Std. Deviation
CaseR	623.800	4528	2454.100	2366.400	894.898
DeathR	0	708.900	197.300	206.000	110.145
%Old	0	0.293	0.124	0.132	0.048
%White	0.008	0.947	0.362	0.366	0.267
%NoCit	0.003	0.397	0.149	0.159	0.074
Income	21,600	250,001	60,500	67,152	33,516.460
%FS	0	0.546	0.151	0.183	0.133
%NoHS	0.004	0.488	0.157	0.172	0.105
AsthER	16.700	317.400	65.800	95.570	77.703
FPM	6.100	10.300	7.100	7.430	0.979
Ozone	24	35.200	30.300	29.850	2.263
%Obese	0.011	0.449	0.240	0.246	0.086
%Crowd	0.021	0.990	0.079	0.122	0.169
%Pov	0.062	0.414	0.150	0.178	0.088
Grocery	0.490	0.930	0.820	0.781	0.124
%Dis	0.056	0.111	0.061	0.067	0.018
%NoIns	0.053	0.101	0.078	0.081	0.017
Mobility	2.700	3.600	3.300	3.184	0.297
%DecT	0.330	0.630	0.520	0.498	0.098

**Table 4:** Correlation matrix of all explanatory variables and response variables, containing correlation values in the upper triangle and *p*-values in the lower triangle.

	**%Old**	**%White**	**%NoCit**	**Income**	**%FS**	**%NoHS**	**AsthER**	**FPM**	**Ozone**	**%Obese**	**%Crowd**	**%Pov**	**Grocery**	**%Dis**	**%NoIns**	**Mobility**	**%DecT**	**CaseR**	**DeathR**
**%Old**	1	0.317	-0.405	0.018	−0.288	−0.303	−0.338	−0.213	0.192	−0.208	0.042	−0.0370	−0.121	−0.080	0.086	0.074	0.042	0.129	0.0270
**%White**	<0.001 *	1	−0.450	0.652	−0.686	−0.732	−0.553	0.309	−0.377	−0.548	−0.074	−0.490	−0.109	−0.294	−0.388	−0.243	0.325	−0.532	−0.498
**%NoCit**	<0.001 *	<0.001 *	1	−0.244	0.296	0.532	0.017	0.161	−0.047	0.031	0.074	0.241	0.096	0.026	0.233	−0.029	0.037	0.088	0.159
**Income**	0.81	<0.001 *	0.001 *	1	−0.756	−0.768	−0.499	0.427	−0.505	−0.586	−0.267	−0.631	−0.129	−0.30	−0.295	−0.374	0.504	−0.544	−0.503
**%FS**	<0.001 *	<0.001 *	<0.001 *	<0.001 *	1	0.860	0.698	−0.197	0.283	0.647	0.293	0.817	0.357	0.503	0.058	0.125	−0.511	0.422	0.443
**%NoHS**	<0.001 *	<0.001 *	<0.001 *	<0.001 *	<0.001 *	1	0.516	−0.268	0.372	0.563	0.186	0.728	0.209	0.388	0.270	0.256	0.464	0.480	0.459
**AsthER**	<0.001 *	<0.001 *	0.83	<0.001 *	<0.001 *	<0.001 *	1	−0.031	0.104	0.661	0.150	0.826	0.352	0.545	−0.075	−0.054	−0.376	0.240	0.235
**FPM**	0.004 *	<0.001 *	0.03 *	<0.001 *	0.009 *	0.009 *	0.68	1	−0.881	−0.487	−0.144	−0.047	0.356	−0.01	−0.444	−0.721	0.455	−0.600	−0.464
**Ozone**	0.011 *	<0.001 *	0.53	<0.001 *	<0.001 *	<0.001 *	0.17	<0.001 *	1	0.550	0.222	0.141	−0.171	0.077	0.687	0.747	−0.535	0.643	0.561
**%Obese**	0.005 *	<0.001 *	0.68	<0.001 *	<0.001 *	<0.001 *	<0.001 *	<0.001 *	<0.001 *	1	0.253	0.634	0.021	0.455	0.264	0.484	−0.640	0.518	0.370
**%Crowd**	0.58	0.33	0.33	<0.001 *	<0.001 *	<0.001 *	0.047 *	0.055* *	0.003 *	<0.001 *	1	0.268	0.342	0.005	0.037	0.059	−0.228	0.052	0.167
**%Pov**	<0.001 *	<0.001 *	0.001 *	<0.001 *	<0.001 *	<0.001 *	<0.001 *	0.54	0.06* *	<0.001 *	<0.001 *	1	0.417	0.539	−0.026	0.024	−0.475	0.245	0.220
**Grocery**	0.11	0.15	0.21	0.088* *	<0.001 *	<0.001 *	<0.001 *	<0.001 *	0.02 *	0.78	<0.001 *	<0.001 *	1	0.328	−0.157	−0.498	−0.169	−0.233	0.0002
**%Dis**	0.29	<0.001 *	0.73	<0.001 *	<0.001 *	<0.001 *	<0.001 *	0.91	0.31	<0.001 *	0.63	<0.001 *	<0.001 *	1	0.010	0.102	−0.697	0.358	0.168
**%NoIns**	0.25	<0.001 *	0.002 *	<0.001 *	0.446	<0.001 *	0.32	<0.001 *	<0.001 *	<0.001 *	0.63	0.73	0.04 *	0.89	1	0.621	−0.292	0.416	0.354
**Mobility**	0.33	0.001 *	0.71	<0.001 *	0.098* *	<0.001 *	0.48	<0.001 *	<0.001 *	<0.001 *	0.43	0.75	<0.001 *	0.18	<0.001 *	1	−0.665	0.599	0.339
**%DecT**	0.57	<0.001 *	0.62	<0.001 *	<0.001 *	<0.001 *	<0.001 *	<0.001 *	<0.001 *	<0.001 *	0.002 *	<0.001 *	0.02 *	<0.001 *	<0.001 *	<0.001 *	1	−0.545	−0.328
**CaseR**	0.09* *	<0.001 *	0.24	<0.001 *	<0.001 *	<0.001 *	0.001 *	<0.001 *	<0.001 *	<0.001 *	0.49	0.001 *	0.002 *	<0.001 *	<0.001 *	<0.001 *	<0.001 *	1	0.7422
**DeathR**	<0.001 *	<0.001 *	0.04 *	<0.001 *	<0.001 *	<0.001 *	0.002 *	<0.001 *	<0.001 *	<0.001 *	0.03 *	0.003 *	0.998	0.025 *	<0.001 *	<0.001 *	<0.001 *	<0.001 *	1

* Significant at 0.05 level; ** Significant at 0.10 level.

**Table 5:** Multiple regression of predicting New York City COVID-19 case rates per 100,000 residents, both non-stratified and stratified by borough.

	Non-Stratified			Stratified														
	Overall			Manhattan			Staten Island			Bronx			Queens			Brooklyn		
	B	*SE*	Sig	B	*SE*	Sig	B	*SE*	Sig	B	*SE*	Sig	B	*SE*	Sig	B	*SE*	Sig
Constant	−576	1446	0.69	490.8	1421	0.73	9831	6839	0.22	7995	6874	0.26	−4039	2278	0.08	−8811	2263	<0.001
%Old	3204	1070	0.003	3017	1440	0.04	−1148	4420	0.81	1697	5270	0.75	1702	2393	0.48	728.4	2300	0.75
%White	−1315	272.7	<0.001	−519	1063	0.63	63.47	1934	0.98	−2215	891	0.02	−1715	431	<0.001	439.9	572	0.45
%NoCit	564.8	784.0	0.47	4200	1414	0.01	2210	4846	0.67	323.1	2837	0.91	−2279	1285	0.08	−6423	1352	0.64
%FS	517.3	597.1	0.39	1041	1899	0.59	3955	6040	0.55	−4520	1834	0.03	−185	1623	0.91	4282	822	<0.001
Ozone	176.4	37.26	<0.001	−8.34	61.2	0.89	−232	259	0.42	−124	217	0.58	221.8	78.1	0.01	300.2	70.2	<0.001
AsthER	−0.72	1.050	0.49	2.124	1.67	0.21	−2.23	6.83	0.76	1.161	4.07	0.78	8.428	4.02	0.04	−4.24	2.18	0.06
%Obese	−791	937.8	0.40	1423	1516	0.35	−1669	1701	0.38	611.5	5164	0.91	−4475	1625	0.01	5041	2521	0.06
%Crowd	13.85	291.8	0.96	−2073	3954	0.60	+			2017	3982	0.62	12,665	2247	<0.001	−987	312	0.004
Grocery	−2318	424.8	<0.001	+			+			+			+			+		
%Dis	12,976	4259	0.003	+			+			+			+			+		
%NoIns	−7807	3908	0.05	+			+			+			+			+		
%DecT	−1220	871	0.16	+			+			+			+			+		

^+^ Variable not included due to singularities. B: unstandardized beta, SE: standard error, Sig: significance level

**Table 6:** Multiple regression of predicting New York City COVID-19 death rates per 100,000 residents, both non-stratified and stratified.

	Non-Stratified			Stratified														
	Overall			Manhattan			Staten Island			Bronx			Queens			Brooklyn		
	B	*SE*	Sig	B	*SE*	Sig	B	*SE*	Sig	B	*SE*	Sig	B	*SE*	Sig	B	*SE*	Sig
Constant	−488	204	0.02	−281	222.1	0.21	120.7	1364	0.93	2611	1289	0.06	−839.1	351.7	0.02	−328	295.0	0.28
%Old	1006	151	<0.001	714	225.0	0.003	311.6	882	0.74	1619	988	0.12	1152	369.4	0.003	1993	299.9	<0.001
%White	−119	38	0.002	40.5	166.1	0.81	137.9	386	0.74	−522	167	0.01	−66.42	66.56	0.32	−71.8	74.61	0.34
%NoCit	313	111	0.005	649	221.0	0.006	196.8	967	0.85	229	532	0.67	410.3	198.4	0.04	62.46	176.3	0.73
%FS	174	84.3	0.04	226	296.8	0.45	1925	1205	0.19	−416	344	0.24	501.9	250.5	0.05	359.8	107.1	0.002
Ozone	22.9	5.26	<0.001	4.17	9.566	0.67	−2.18	51.7	0.97	−85	40.8	0.06	24.36	12.05	0.05	5.993	9.145	0.52
AsthER	0.20	0.15	0.19	0.55	0.261	0.04	−0.74	1.36	0.61	−0.7	0.76	0.36	1.236	0.621	0.05	0.054	0.284	0.85
%Obese	−178	132	0.18	22.7	236.9	0.92	−833	339	0.07	1283	968	0.20	−369.1	250.9	0.15	256.7	328.6	0.44
%Crowd	−15	41.2	0.71	106	618.0	0.87	+			−705	746	0.36	583.2	346.9	0.099	−102	40.65	0.02
Grocery	−39	60.0	0.51	+			+			+			+			+		
%Dis	−232	601	0.70	+			+			+			+			+		
%NoIns	−920	552	0.097	+			+			+			+			+		
%DecT	−22	123	0.86	+			+			+			+			+		

^+^ Variable not included due to singularities.

## Data Availability

Publicly available datasets were analyzed in this study. This data can be found here: https://www1.nyc.gov/site/doh/covid/covid-19-data.page (accessed on 5 August 2021); https://statisticalatlas.com/place/New-York/New-York/Overview (accessed on 5 August 2021); https://www1.nyc.gov/site/coronavirus/get-tested/covid-19-testing.page (accessed on 5 August 2021); https://www.census.gov/quickfacts/fact/table/newyorkcitynewyork,kingscountybrooklynboroughnewyork,newyorkcountymanhattanboroughnewyork,queenscountyqueensboroughnewyork/PST045219 (accessed on 5 August 2021); https://www.ers.usda.gov/foodatlas/ (accessed on 5 August 2021); https://covid.cdc.gov/covid-data-tracker/#datatracker-home (accessed on 5 August 2021).
